# Study on the rupture surface morphology and ultimate bearing capacity of a self-anchored test pile

**DOI:** 10.1038/s41598-022-20887-0

**Published:** 2022-09-30

**Authors:** Lin Zhu, Hailong Ma

**Affiliations:** Zhejiang Sci-Tech University, Hangzhou, 310018 China

**Keywords:** Civil engineering, Hydrogeology

## Abstract

The expression of the ultimate bearing capacity of the upper uplift pile and the lower compressive pile of a self-anchored test pile was obtained by studying their rupture surface morphology. The upper uplift pile had a composite shear rupture surface shape, and the lower compressive pile had the Meyerhof rupture surface shape. Since the interaction between the upper pile and lower pile of a self-anchored test pile is negligible, the expression form of the ultimate bearing capacity of a self-anchored test pile was obtained based on the transformation formula of its bearing capacity. Under the test conditions, the rupture surface morphology of a self-anchored test pile belongs to the situation when the equilibrium point is inside the rupture surface of the lower compressive pile. The theoretical rupture surface is approximately 0.09 m away from the pile side at ground level (1.8 d, where d is the pile diameter). Compared with the distance of the measured rupture surface of the upper uplift pile to the pile side, the difference value is -2.17%. The calculated ultimate bearing capacities of the upper uplift and lower compressive piles are 1287.34 N and 1201.65 N, respectively. The ultimate bearing capacity of the self-anchored test pile is approximately 2726.16 N. Compared with the experimental values of the upper pile and lower pile of the self-anchored test pile, the difference values are + 0.97% and − 7.57%, respectively. Compared with the experimental values of the traditional test piles, the difference value is − 2.64%. The rupture surface morphology and the expression of the ultimate bearing capacity of the self-anchored test pile in this paper can provide a research basis reference for calculating the ultimate bearing capacity of the self-anchored test piles with different pile sizes and soil properties.

## Introduction

**Figure 1:**
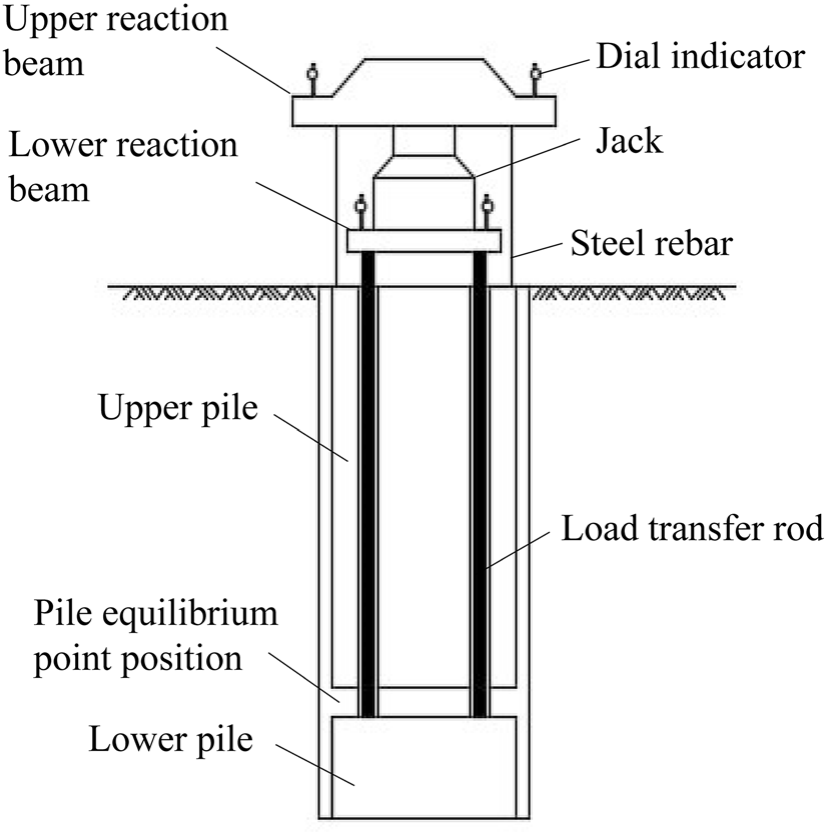
Diagrammatic diagram of a self-anchored test pile.

The most reliable and direct method to test the vertical compressive capacity of a single pile is the vertical compressive static load test, also known as the traditional pile test^1–3^. Due to the disadvantages of complex operation, high cost and low safety, the traditional pile test gradually fails to satisfy the test requirements of piles with large bearing capacity^4^. As a new test pile that was improved based on the reaction system of traditional test piles, the self-anchored test pile uses the self-bearing capacity of piles to provide mutual force^5–8^, and has the advantages of safety, effectiveness, time-savings, simple operation, fewer site restrictions and lower test costs. A diagrammatic diagram of the self-anchored test pile is shown in Fig. 1.

Chen et al.^5^ verified that the hyperbolic function model could be applied to the evolution law of the pile friction and load transfer process of a self-anchored test pile through in-situ tests. Zhou^6^ compared and analyzed three conversion methods through indoor model tests and believed that the simple conversion method was suitable for converting the load–displacement curve of a self-anchored test pile to that of a traditional test pile. Xu and Ma^7^ compared and analyzed four loading types of piles through numerical tests. Based on the principle of load equality and displacement conversion coefficient, the conversion formula of the *Q*–*s* curve from the self-anchored test pile to the traditional test pile was established. Ma et al.^8^ compared the additional stress test in soil by separately loading the upper uplift pile, separately loading the lower compress pile and loading the self-anchored test pile in indoor model tests. The result indicated that the additional stress ratio is very small and the interaction between the upper pile and lower pile of the self-anchored test pile can be negligible.

Meyerhof ^9^ assumed that the soil was a rigid-plastic body and believed that the rupture zone would curl upwards in a pear-shaped shape when the foundation was subjected to vertical compression with increasing burial depth, which is consistent with the actual deformation and rupture of deep foundations. He established a formula to calculate the ultimate end resistance of a deep foundation. Based on Terzaghi, Zhou et al.^10^ deduced the bearing capacity of a circular foundation considering the space effect by using the static balance method. Zhang et al.^11^ compared the slip line method and the multi-block upper limit method of ultimate bearing capacity of foundation and verified the correctness and wide applicability of Meyerhof’s unified solution of ultimate bearing capacity. Comparing Meyerhof’s solution and Zhang’s solution, Li et al.^12^ considered the influence of width, depth-to-width ratio, wall friction, matric suction, and soil parameter adjustment coefficient on the bearing capacity of the foundation, and proposed a solution to the bearing capacity of unsaturated soil strip foundation. Chattopadhyay and Pise^13^ proposed a method to calculate the ultimate bearing capacity of uplift piles assuming that the rupture surface of the soil around piles was a curve-shaped composite rupture surface. Qian et al.^14^ conducted an indoor test of the uplift anchor plate, and the results showed that the rupture surfaces on both sides of the horizontal uplift anchor plate were trumpet-shaped under the vertical load, and the tangent of the rupture surface was approximately vertical at the edge of the plate. He et al.^15^ discussed the relationship between ultimate bearing capacity, fracture surface and soil property of uplift pile in the layered foundation by applying the horizontal strip method and limit equilibrium principle. Faizi et al.^16^ obtained that the deformation mechanism of the uplift pile is similar to a curve shape through the model test of the semi-circular section pile. Wang et al.^17^ deduced the calculation method of ultimate uplift bearing capacity of the rock-socketed pedestal pile overburden soil according to the results of the centrifugal model test and limit equilibrium method, and obtained that the soft rock failure mode of the pile was horn shaped surface, which was described by a unified power function form.

At present, there are few reports on the bearing capacity characteristics of self-anchored test piles through the rupture surface morphology. In this paper, the rupture surface shapes of the upper pile and lower pile of a self-anchored test pile are analyzed, and the expression form of the ultimate bearing capacity of the rupture surface of the upper and lower pile is deduced. Simultaneously, two rupture surface morphologies of a self-anchored test pile are obtained according to their position of the equilibrium point. The interaction effect between the upper and lower sections of a self-anchored test pile could be ignored^8^. The ultimate bearing capacity expression can be established through the transformation formula of the bearing capacity of the self-anchored test pile which is according to its rupture surface morphology. The ultimate bearing capacity calculated by the formula established in this paper is close to that obtained by the indoor model test, which provides a new method to calculate the ultimate bearing capacity of a self-anchored test pile.

## Rupture surface morphology and ultimate bearing capacity expression form of the upper section and lower section of a self-anchored test pile

### Rupture surface morphology and ultimate bearing capacity expression form of the upper uplift pile

#### Fundamental assumption

1. There is stress concentration exists at the pile tip under ultimate uplift load, so the rupture surface of the upper uplift pile is tangent to the surface of the pile tip.
2. The included angle of the rupture surface of the upper uplift pile between the Earth’s surface and the horizontal surface is (45° − φ/2)^18,19^.

#### Expression form of the ultimate bearing capacity of the upper uplift pile rupture surface

**Figure 2:**
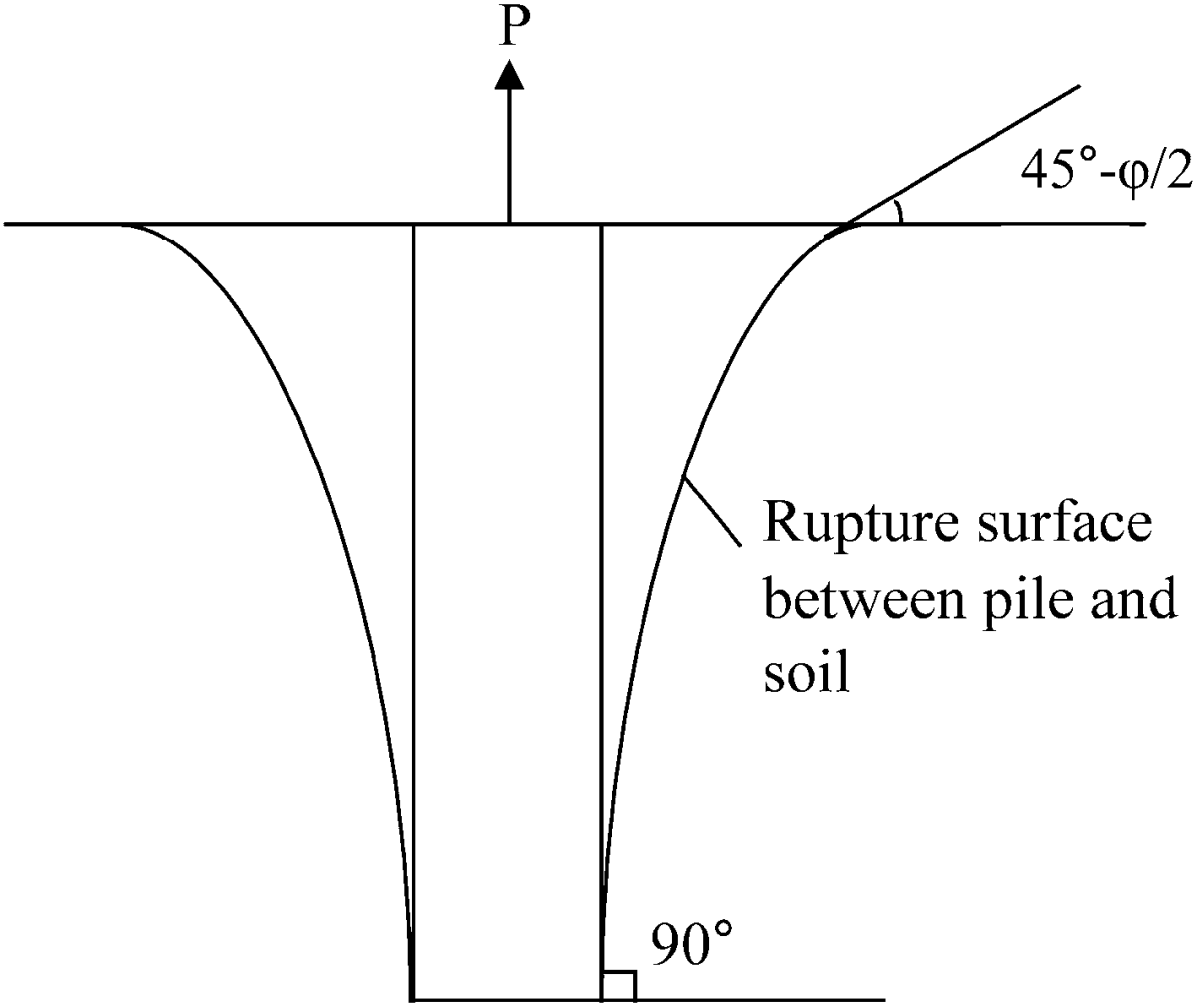
Composite shear rupture surfaces of the upper uplift pile.

The rupture surface of the upper uplift pile adopts the shape of a composite shear rupture surface^13^, as shown in Fig. 2. According to the above two-point assumptions and Fig. 2, the rupture surface of the upper uplift pile of the self-anchored test pile can be described by a differential equation as shown in Eq. (1):

1 $$\frac{{d}_{z}}{{d}_{x}}=\mathrm{tan}\left(45^\circ -\varphi /2\right){\left(\frac{L}{z}\right)}^{N}$$

where $\varphi$ is the internal friction angle of the soil; L is the length of the upper uplift pile; N is a dimensionless and undetermined parameter.

Integrating Eq. (1) and according to the boundary conditions: $x_{{(z=0)}}= {r}$, the parameter equation of the rupture surface used to describe the upper uplift pile can be obtained by substituting, as shown in Eq. (2):

2 $$x=r+\frac{L}{\mathrm{tan}\left(45^\circ -\varphi /2\right)\left(N+1\right)}{\left(\frac{z}{L}\right)}^{\left(N+1\right)}$$

where, *r* is the radius of the pile.

**Figure 3:**
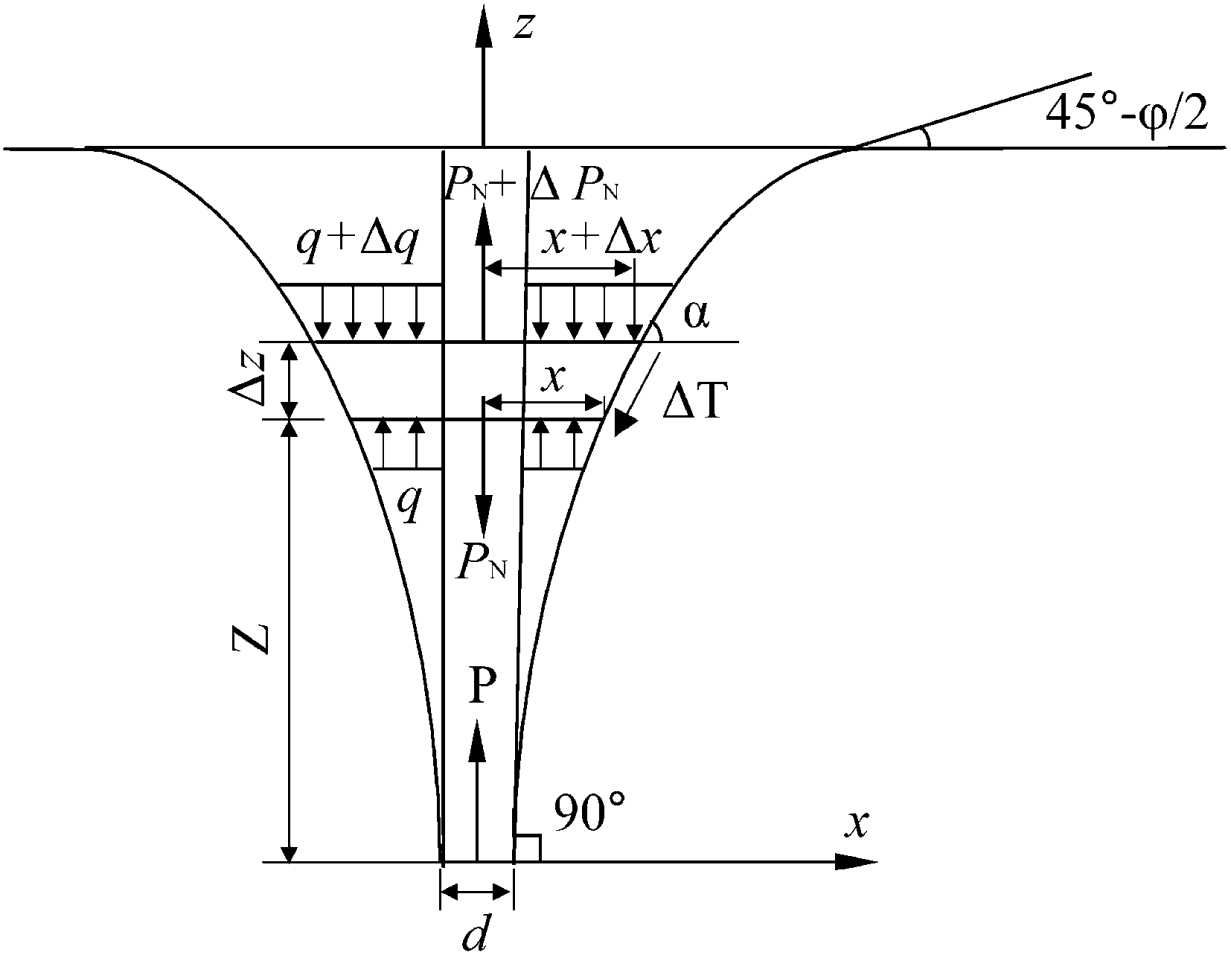
Calculation model of the ultimate uplift bearing capacity of the upper uplift pile.

Figure 3 is a calculation model of the ultimate uplift bearing capacity of the upper uplift pile. The element of the failure soil is taken for limit equilibrium analysis in Fig. 3. According to the Moore–Coulomb criterion, horizontal and vertical balance conditions of the element body and extreme value principle, the expression form of the ultimate bearing capacity of the uplift pile in the upper section of a single-layer foundation is shown in Eq. (3)^20,21^:

3 $$P = \pi d\gamma L\int_{0}^{L} {\frac{{2x}}{d}\left[ {\left( {1 - \frac{z}{L}} \right)\cot \alpha + \left( {1 - \frac{z}{L}} \right)\left( {\cos \alpha + K\sin \alpha } \right) \cdot \left( {\tan \varphi + \cot \alpha } \right) + \frac{c}{{\gamma L}} + \frac{x}{{2L}}} \right]d_{Z} }$$

where *γ* is the soil weight; *α* is the included angle between the tangent line of the rupture surface about the uplift pile and the horizontal plane, $\mathrm{tan}\alpha =\frac{{d}_{z}}{{d}_{x}};\mathrm{sin}\alpha =\sqrt{\frac{{\mathrm{tan}}^{2}\mathrm{\alpha }}{1+{\mathrm{tan}}^{2}\mathrm{\alpha }}}$; *K* is the lateral pressure coefficient of the soil, $K=\frac{\mu }{1-\mu }$, *μ* is the Poisson ratio; $c$ is the cohesion of the soil around the pile.

By substituting Eq. (2) into Eq. (3), we obtain the equation for N. There may be many rupture surfaces, among which the real rupture surface of the uplift pile has the smallest uplift load. The dimensionless and undetermined parameter N can be determined according to the extreme value principle^22^ of the rupture surface for the minimum uplift load, as shown in Eq. (4):

4 $$\frac{dP}{dN}=0,\frac{{d}^{2}P}{{d}^{2}N}\le 0$$

### Rupture surface morphology and ultimate bearing capacity expression form of the lower compressive pile

#### Fundamental assumption

1. Assuming that the rupture surface extends to the surface, use the normal stress and tangential stress on the "equivalent free surface" to reflect the resultant force on the side of the pile foundation and the gravity of the nearby soil.
2. The foundation soil is homogeneous unsaturated soil and the soil on the sliding surface is in a state of plastic limit equilibrium, which satisfies the unified solution of the shear strength of the unsaturated soil^23^.

#### Expression form of the ultimate bearing capacity of the lower compressive pile rupture surface

**Figure 4:**
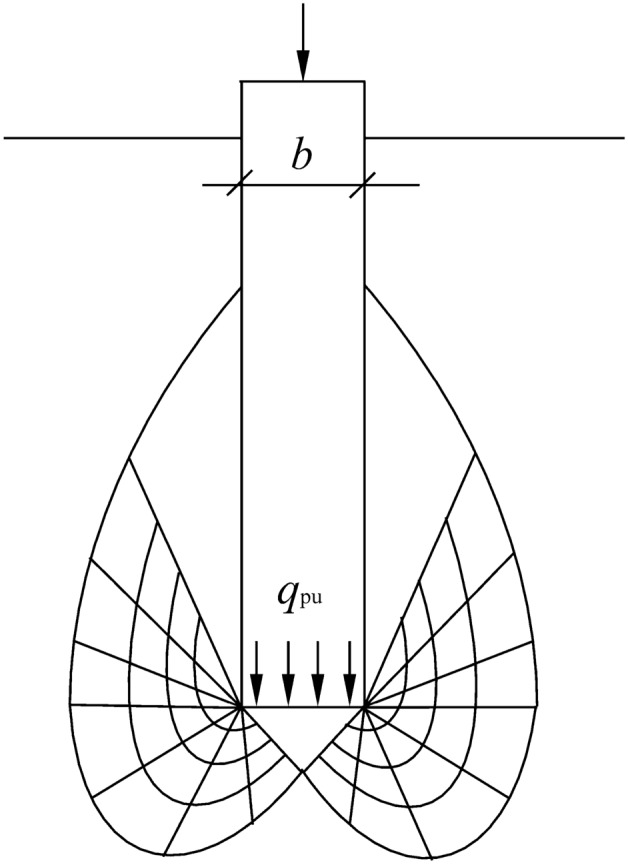
Meyerhof rupture surface of the lower compressive pile.

The rupture surface shape of the lower compressive pile of a self-anchored test pile conforms to the Meyerhof rupture surface shape, as shown in Fig. 4. According to the ultimate bearing capacity theory, the expression of the ultimate bearing capacity of the lower compressive pile is shown in Eq. (5):

5 $${q}_{\mathrm{pu}}= \frac{1}{2}\gamma b{N}_{\upgamma }+q{N}_{\mathrm{q}} +c{N}_{\mathrm{c}}$$

where, ${N}_{\upgamma }$, ${N}_{\mathrm{q}}$, and ${N}_{\mathrm{c}}$ are the coefficients of bearing capacity, and their values are only related to the $\varphi$; *b* is the width or diameter of the pile; *q* is the vertical dead weight stress in the soil at the elevation of the pile bottom, and $q=\gamma$*L*; *c* is the cohesion of the soil.

## Rupture surface morphology of a self-anchored test pile

**Figure 5:**
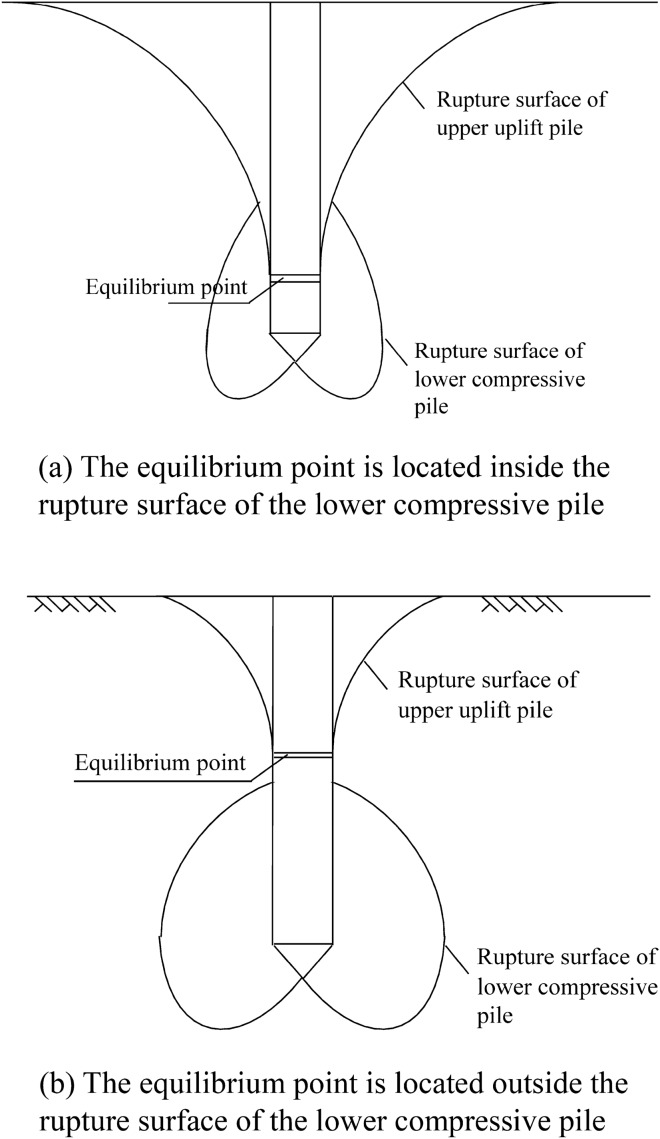
Rupture surface morphology of the self-anchored test pile.

The self-anchored test pile is divided into upper uplift pile and lower compressive pile at the equilibrium position of the pile body force. The upper section of the self-anchored test pile is subjected to an uplift force, and the rupture surface is a composite shear rupture surface. The lower section of the self-anchored test pile is subjected to pressure at the top of the pile, and the rupture surface is the Meyerhof rupture surface. The interaction between the upper pile and lower pile of the self-anchored test pile can be negligible^8^, therefore, the rupture surface morphology of the self-anchored test pile is independently formed by the rupture surface of the upper uplift pile and lower compressive pile and not affected by the other pile. According to the rupture surface morphology of the upper uplift pile and lower compressive pile and combined with the position of the equilibrium point, the following two rupture surface morphologies of the self-anchored test pile can be constructed, as shown in Fig. 5.

When the equilibrium point position is located inside the rupture surface of the lower compressive pile, as shown in Fig. 5a, the upwards crimping of the Meyerhof rupture surface of the lower compressive pile stops when it intersects the composite shear rupture surface of the upper uplift pile. When the equilibrium point position is located outside the rupture surface of the lower compressive pile, as shown in Fig. 5b, the rupture surfaces of the upper uplift pile and lower compressive pile are separately formed without intersection.

## Expression of the ultimate bearing capacity of the self-anchored test pile

**Figure 6:**
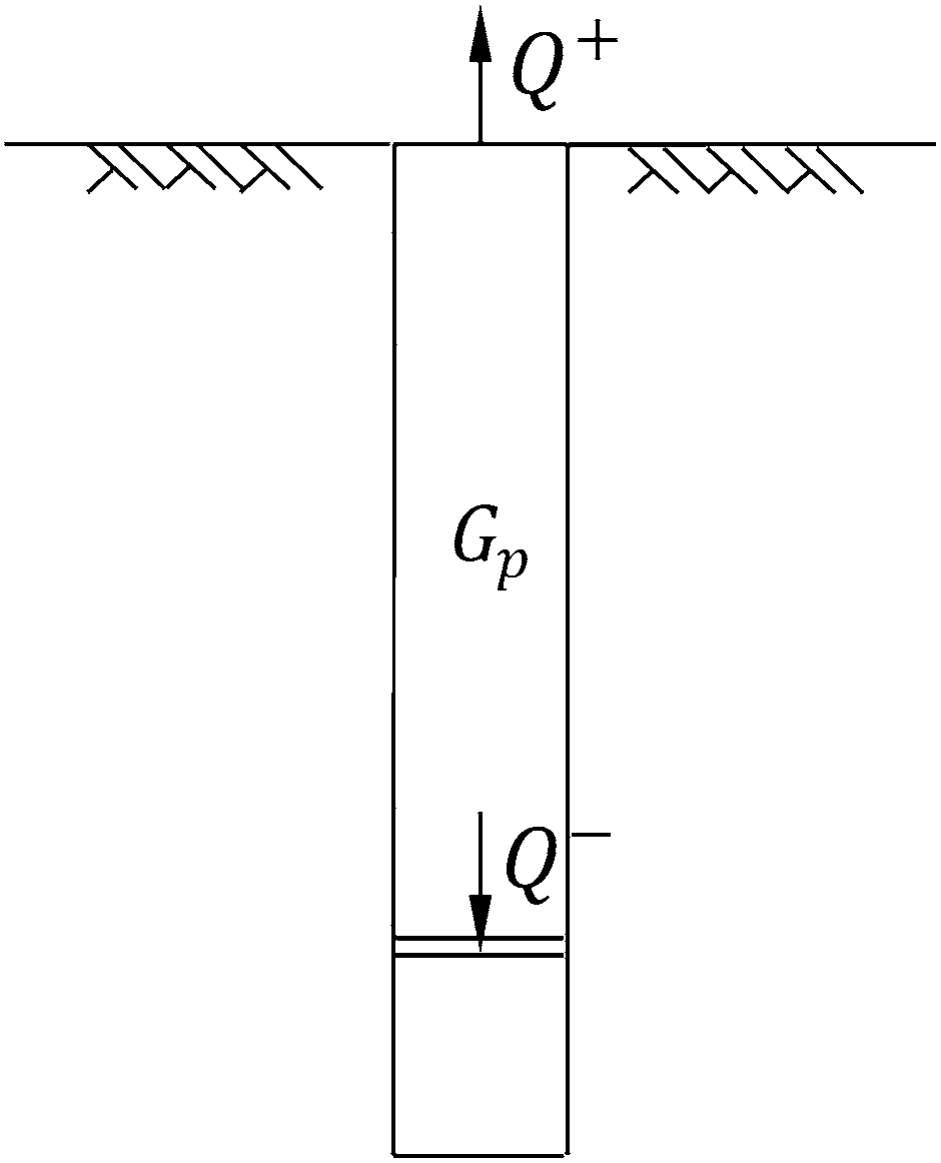
Schematic diagram of the ultimate bearing capacity of a self-anchored test pile.

Because the interaction effect between the upper pile and lower pile can be ignored^8^, the ultimate bearing capacity of the self-anchored test pile is superimposed by the ultimate bearing capacity of the upper uplift pile and lower compressive pile. However, the upper uplift pile bears the upward tension, and the lower compressive pile bears the downward pressure, as shown in Fig. 6. Therefore, it is necessary to convert the ultimate bearing capacity of uplift into that of compression. The conversion formula of the bearing capacity of a self-anchored test pile^7^ is shown in Eq. (6):

6 $${Q}_{u}=\frac{{Q}^{+}-{G}_{P}}{{\lambda }_{1}}+{Q}^{-}$$

where, ${Q}_{u}$ is the bearing capacity of a single pile; ${Q}^{+}$ is the uplift force of the upper uplift pile; ${G}_{P}$ is the gravity of the upper uplift pile; ${\lambda }_{1}$ is the conversion coefficient of positive and negative friction; ${Q}^{-}$ is the pressure of the lower compressive pile.

Substituting the expression of the ultimate bearing capacity of the upper uplift pile obtained from the composite shear rupture surface and that of the lower compressive pile which obtained from the Meyerhof rupture surface into Eq. (6), we obtain the expression of the ultimate bearing capacity of the self-anchored test pile as shown in Eq. (7):

7 $$\begin{aligned} Q_{u} & = \frac{{\int_{0}^{L} {\frac{{2x}}{d}\left[ {\left( {1 - \frac{z}{L}} \right)\cot \alpha + \left( {1 - \frac{z}{L}} \right)\left( {\cos \alpha + K\sin \alpha } \right) \cdot \left( {\tan \varphi + \cot \alpha } \right) + \frac{c}{{\gamma L}} + \frac{x}{{2L}}} \right]d_{Z} } }}{{\lambda _{1} }} \\ & \quad \times \frac{{\pi d\gamma L}}{{\lambda _{1} }} - \frac{{G_{P} }}{{\lambda _{1} }} + \frac{1}{2}\gamma bN_{\lambda } + qN_{q} + cN_{c} \\ \end{aligned}$$

## Indoor model test validation of the theoretical rupture surface morphology and ultimate bearing capacity

To verify the accuracy of the fracture surface morphology and the expression of the ultimate bearing capacity, the indoor model test^6^ was taken as an example to obtain the ultimate bearing capacity of the self-anchored test pile based on the soil properties and pile parameters, and the self-anchored test pile was compared with the traditional test pile in terms of ultimate bearing capacity.

### Indoor model test

#### Model pile

**Figure 7:**
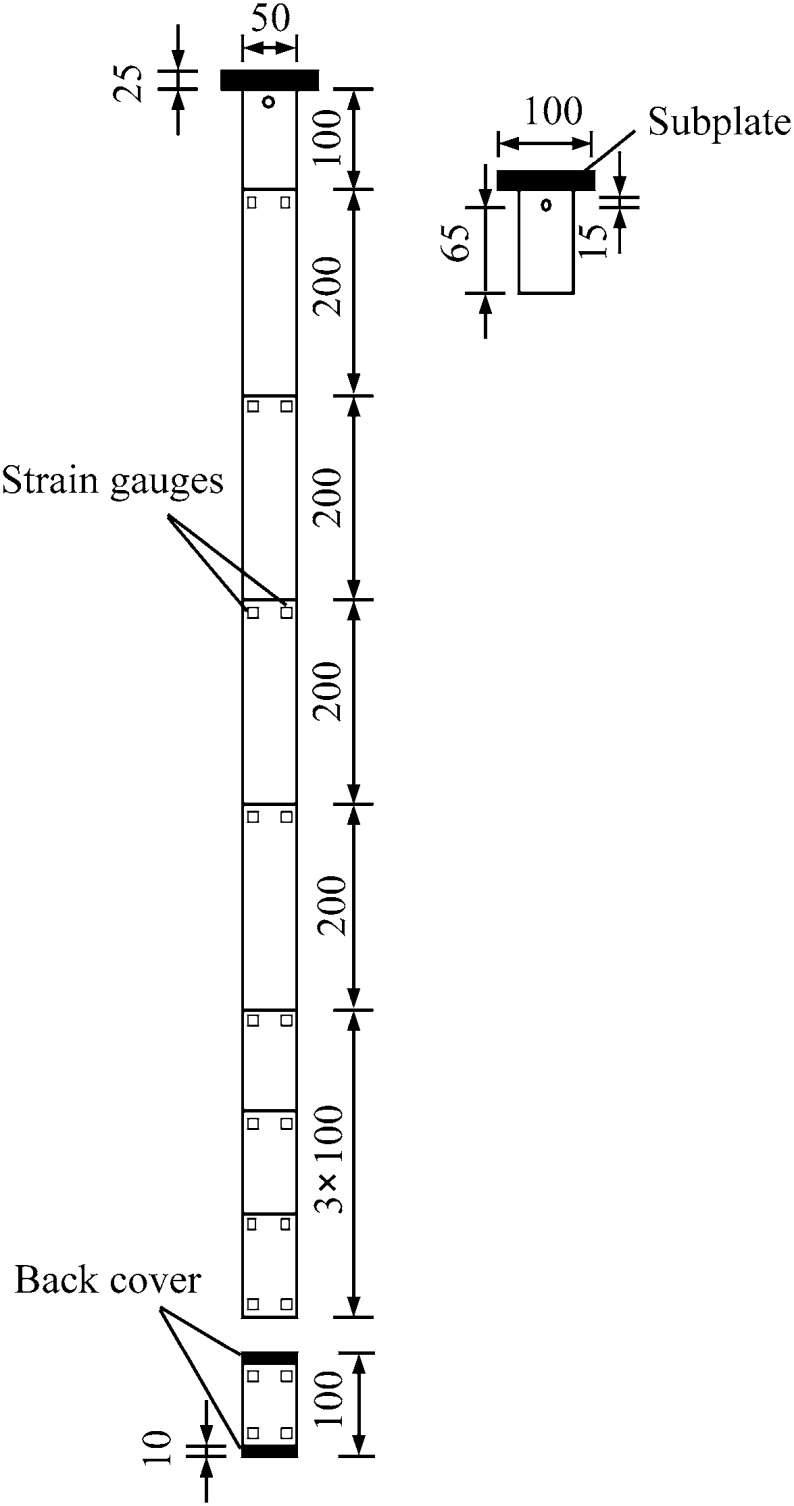
Layout of the strain gauges distribution diagram of a self-anchored test pile.

The model pile is made of plexiglass pipe. The diameter of the model pile is 50 mm, the wall thickness is 8 mm, the length of the upper uplift pile is 1100 mm, the gravity of the upper pile is 22 N, and the length of the lower compressive pile is 100 mm. The upper uplift pile is composed of three 100 mm single-segment piles and four 200 mm single-segment piles, which are splicing by two ends of threads, and the subplate exposing the surface of the soil is 100 mm. The lower compressive pile is a single section pile of 100 mm. The traditional test pile is composed of six 200 mm single-segment piles, which are splicing by two ends of threads, with a total length of 1200 mm. As the plexiglass pipe wall is relatively smooth, a layer of standard sand is uniformly bonded around the model pile to increase the roughness of the pile wall. The BX120-3AA resistance strain gauges are used to detect the strain on the side of the pile, and the connected wires are led out from the pile head. The changes of the strain gauges are recorded automatically through DH3820 collector. The layout of the strain gauges distribution diagram of a self-anchored test pile is shown in Fig. 7.

#### Loading device

**Figure 8:**
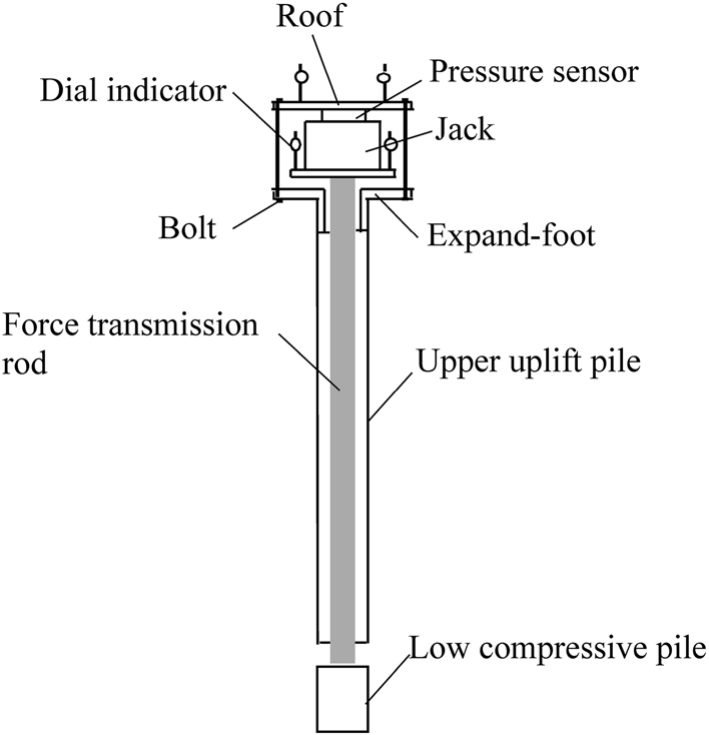
Loading device of a self-anchored test pile.

The loading device of the self-anchored test pile is self-made. When the jack applies the load, the jack and pressure sensor are on the roof, and the pulling force is applied to the upper uplift pile; the bottom of the jack is placed on the disk, and the pressure is transferred to the lower compressive pile through the force transmission rod, so that the same load is applied to the upper and lower sections of the self-anchored pile at the same time, as shown in Fig. 8. The loading of the traditional test pile is carried out by a servo motor, and the loading value and vertical displacement are recorded by computer. The loading value of the self-anchored test pile is measured by the pressure sensor, and the vertical displacement is measured by the dial indicator. The test was carried out by the slow maintenance load method using graded loading in equal amounts step by step.

#### Test soil parameters

Silt was used as test soil in the indoor model test. The physical and mechanical indices of silt are shown in Table 1.

**Table 1:** Physical and mechanical indices of silt.

Density (g/cm^3^)	Water content (ω/%)	Cohesion (c/kPa)	Internal friction angle ($\phi$/°)	Compression modulus (Es/MPa)	Poisson ratio (μ)
1.61	9.8	5	26	8.1	0.25

#### Test results

**Figure 9:**
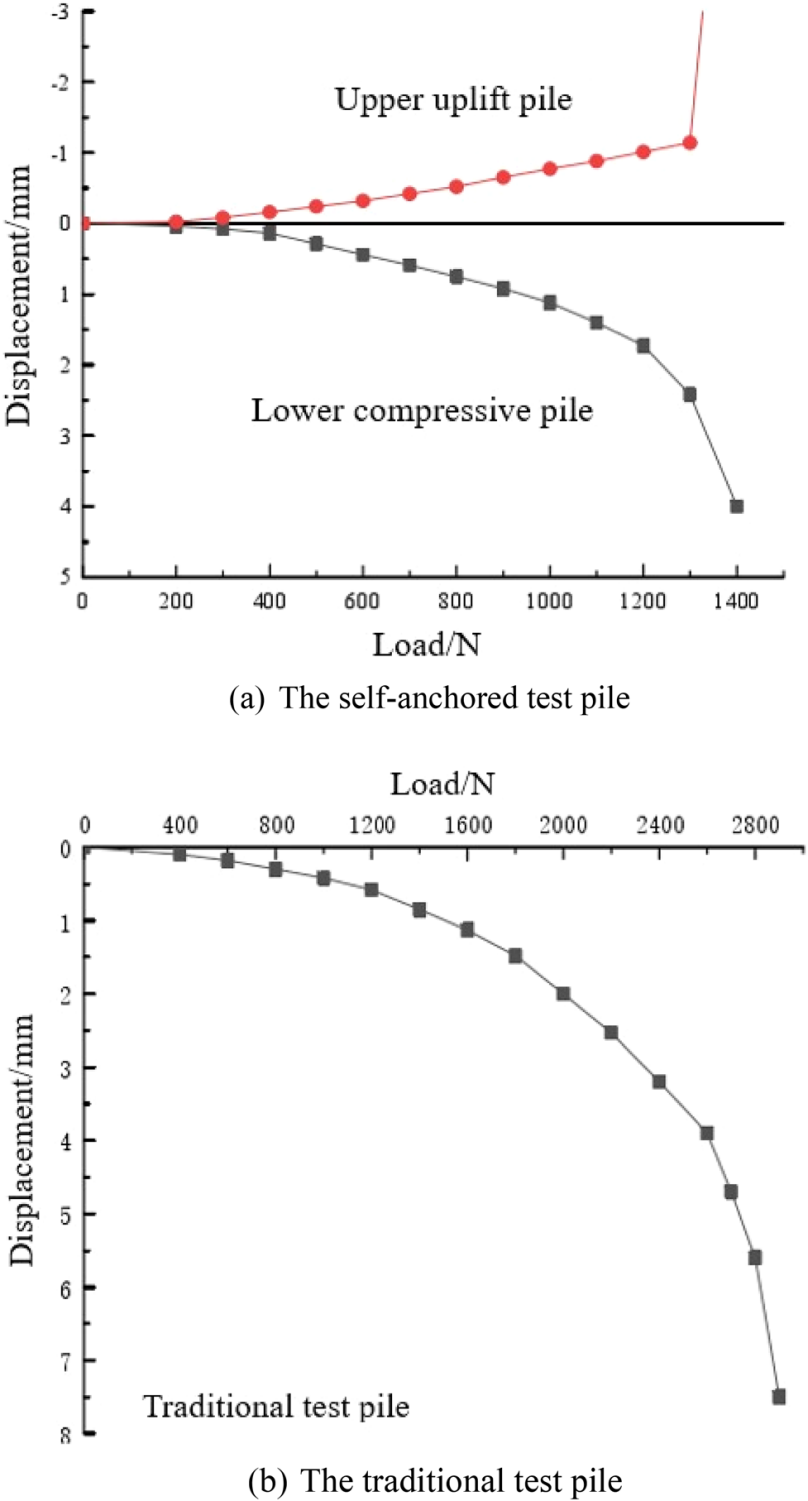
Load–displacement curve.

By recording the pile top displacement of each test pile at different load levels, the load–displacement curve is obtained, as shown in Fig. 9. The load–displacement curve of the self-anchored test pile is divided into upper uplift pile and lower compressive pile. The ultimate bearing capacity of the upper uplift pile and lower compressive pile are both 1300 N. The ultimate displacement of upper uplift pile is 1.14 mm and the ultimate displacement of lower compressive pile is 2.43 mm when the ultimate bearing capacity is reached. The ultimate bearing capacity of the traditional test pile is 2800 N, and the ultimate displacement is 5.6 mm.

#### The measured rupture surface morphology

**Figure 10:**
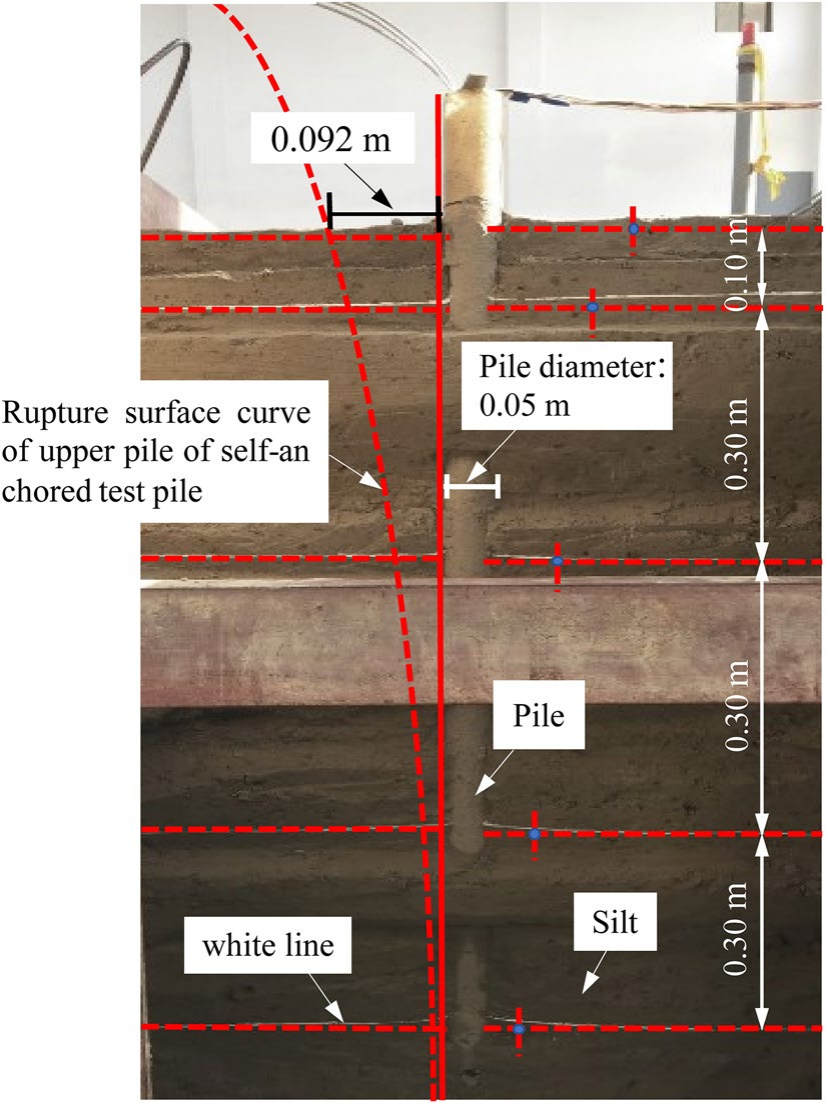
Profile of the measured rupture surface of the upper uplift pile.

To observe the development range of the rupture surface of the self-anchored test pile, a thin layer of white quartz sand is laid at the design depth of 0.10 m, 0.40 m, 0.70 m and 1.00 m as the deformation reference plane when making the test soil. After the end of the self-anchored pile test, the change of the white line at different depths can be observed when the soil is excavated along the pile side. The places where the white line deformation is zero at different depths are connected along the depth to obtain the rupture surface morphology, as shown in Fig. 10, which is the measured rupture surface. As the white line deformation at different depths, the closer it is to the ground, the wider the rupture surface develops, but the white line deformation becomes smaller.

**Figure 11:**
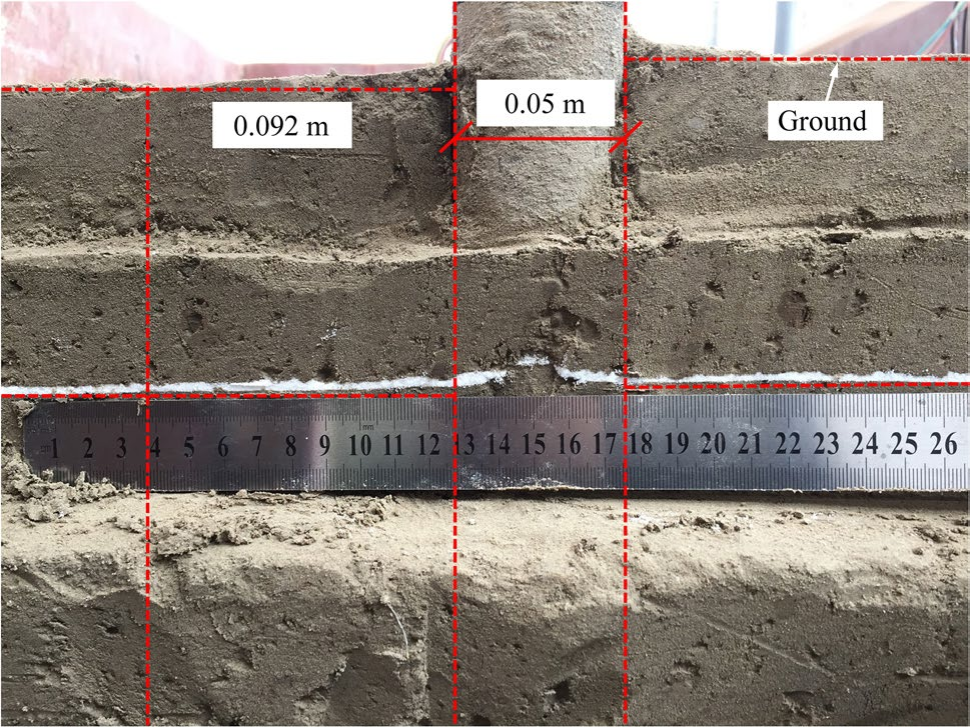
Measured developing range of the upper uplift pile rupture surface on horizontal ground.

The measured rupture surface of the upper uplift pile extends to the ground approximately 0.092 m away from the pile side (1.84 d, where d is the pile diameter), as shown in Fig. 11.

### The theoretical rupture surface morphology

**Figure 12:**
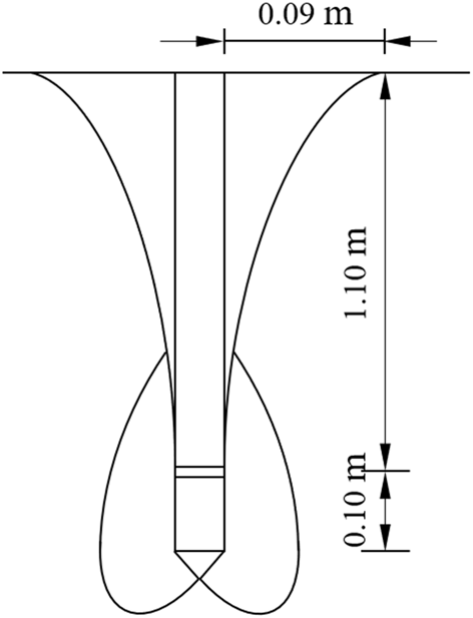
Theoretical rupture surface of a self-anchored test pile.

According to Eq. (2), the theoretical rupture surface is approximately 0.09 m (1.8 d) away from the pile side on the ground, and the difference value is − 2.17% compared to 0.092 m where the measured rupture surface extends to the ground. The theoretical rupture surface of a self-anchored test pile is shown in Fig. 12. The rupture surface of the lower compressive pile intersects that of the upper uplift pile, which belongs to the one case.

### The ultimate bearing capacity of the self-anchored test pile

According to Eq. (3), the ultimate bearing capacity of the upper pile is 1287.34 N. According to the internal friction angle of silt, the bearing capacity coefficients of the pile in the lower section are 0.84, 4.37 and 6.90. According to Eq. (5), the ultimate bearing capacity of the lower compressive pile is 1201.65 N. According to Eq. (7), the ultimate bearing capacity of the self-anchored test pile is 2726.16 N.

Compared with the experimental value, the difference values of the ultimate bearing capacity of the upper uplift and lower compressive piles calculated by the method in this paper are + 0.97% and − 7.57%, respectively. Compared with the ultimate bearing capacity of the traditional test pile, the difference value of the ultimate bearing capacity of a self-anchored test pile calculated by this method is − 2.64%. The results show that the expression of the ultimate bearing capacity of the self-anchored test pile in this paper has a certain rationality and reference value.

## Conclusion

1. In this paper, the rupture surface of the upper uplift pile has a composite shear fracture surface, the rupture surface of the lower compressive pile has the Meyerhof rupture surface. Combined with the equilibrium point, we can infer two types of the rupture surface of the self-anchored test pile. The situation that the equilibrium point is located inside the rupture surface of the lower compressive pile has been verified by the indoor model test. The other case will verify it in future research.
2. If the interaction between the upper uplift and lower compressive piles of the self-anchored test pile is ignored, the rupture surface expression of the ultimate bearing capacity is established based on the transformation formula of the bearing capacity of the self-anchored test pile.
3. The measured rupture surface extends to the ground is approximately 0.092 m away from the pile side (1.84 d, where d is the pile diameter). The closer it is to the ground, the larger the range of the rupture surface is, but the smaller the deformation degree within the range of the fracture surface is.
4. According to the physical and mechanical indices of the soil and pile parameters in the indoor model test, the rupture surface of the self-anchored test pile is the condition that the equilibrium point is located inside the rupture surface of the lower compressive pile. In this case, the rupture surface of the lower compressive pile intersects the rupture surface of the upper uplift pile. The theoretical rupture surface is approximately 0.09 m away from the pile side on the ground (1.8 d), and the difference value is − 2.17% compared to the development range of the measured rupture surface on the ground.
5. Under testing conditions, the ultimate bearing capacity of the upper uplift pile is 1287.34 N, and the ultimate bearing capacity of the lower compressive pile is 1201.65 N. Compared with the experimental values of the upper uplift and lower compressive piles, the difference values are + 0.97% and − 7.57%, respectively. The ultimate bearing capacity of the entire rupture surface of the self-anchored test pile is 2726.16 N, and the difference value is − 2.64% compared to the traditional test pile. Thus, the expression of the ultimate bearing capacity of the self-anchored test pile has good accuracy.

## Data Availability

All data generated or analyzed during this study are included in this published article [and its supplementary information files].
